# Supplementary material for: Contextual expectations in the real-world modulate low-frequency neural oscillations
Source: Imaging Neurosci (Camb). 2025 May 7;3:imag_a_00568. doi: 10.1162/imag_a_00568 (PMC7617707; doi:10.1162/imag_a_00568)
Supplement: Supplementary Material [file imag_a_00568-supp.pdf]

Supplementary Materials for

Contextual Expectations in the Real-World Modulate Low-Frequency Neural Oscillations

Victoria I. Nicholls, *et al*.

Email: nicholls@psych.uni-frankfurt.de

This PDF includes:

Supplementary Figs. 1 to 3.

Supplementary Table 1.

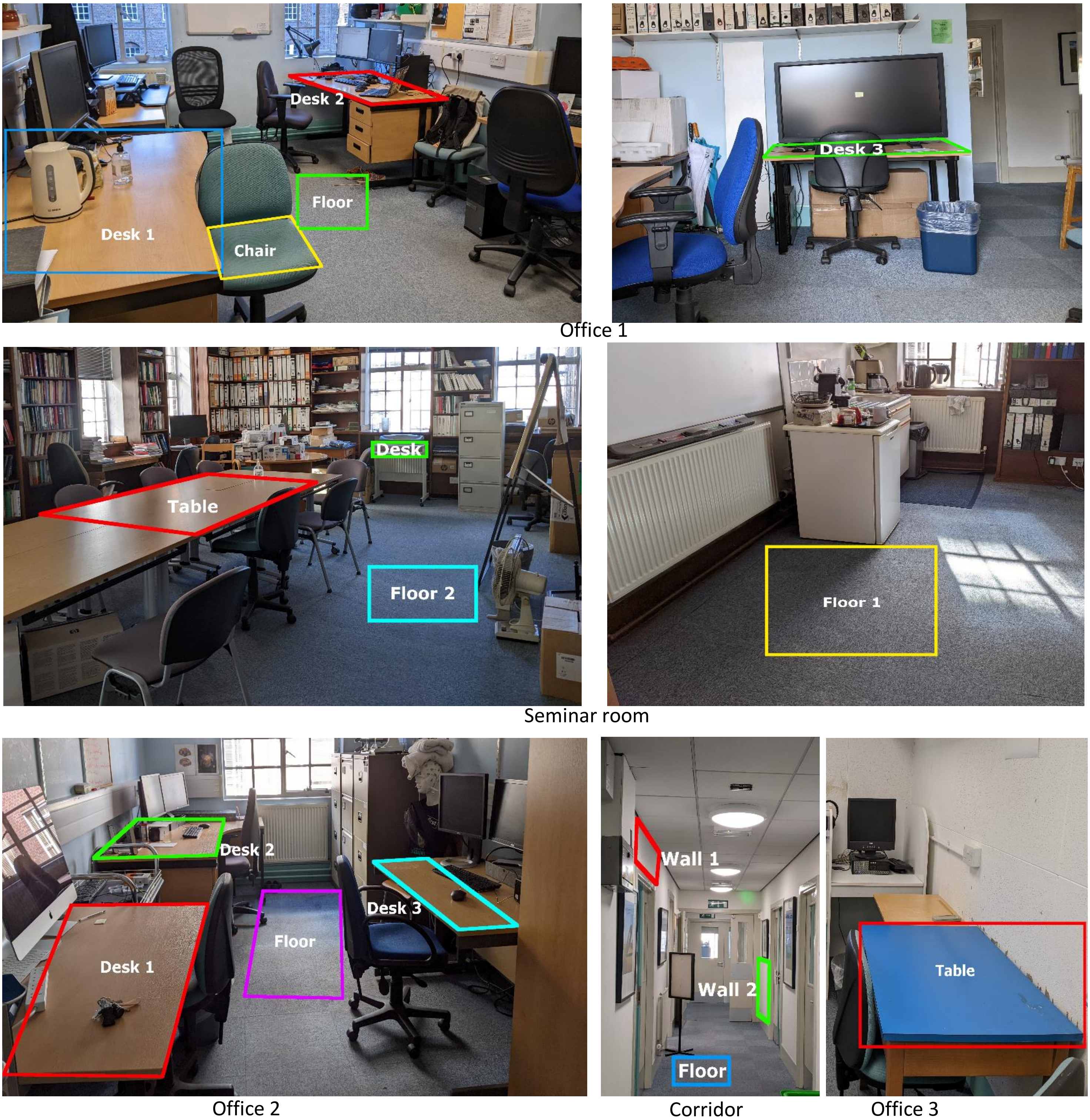

Fig. 1 Objects and their locations in the indoor area of the experiment *The locations in which objects will be placed during the experiment for the indoor environment. The top row shows the locations for Office 1. The second row for the Seminar Room. The bottom row for Office 2, the corridor, and Office 3. See Supplementary Table 1 for which objects will be placed in which locations.*

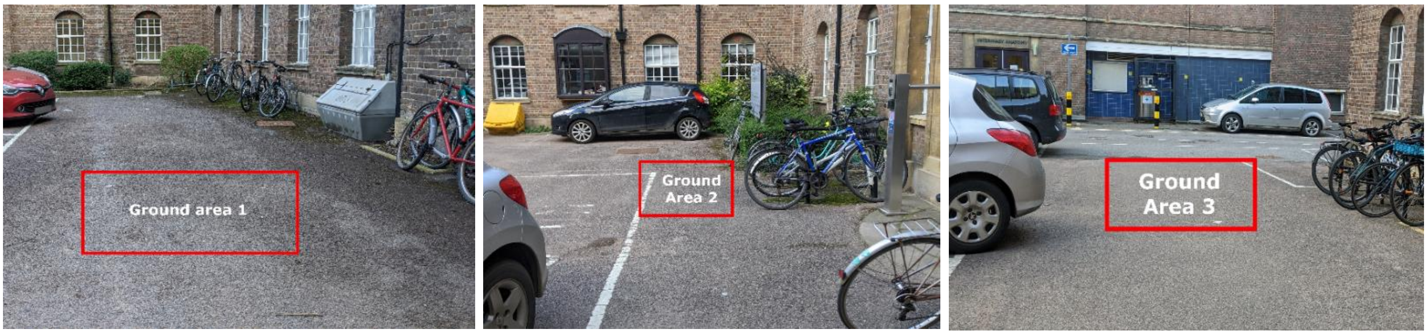

Courtyard

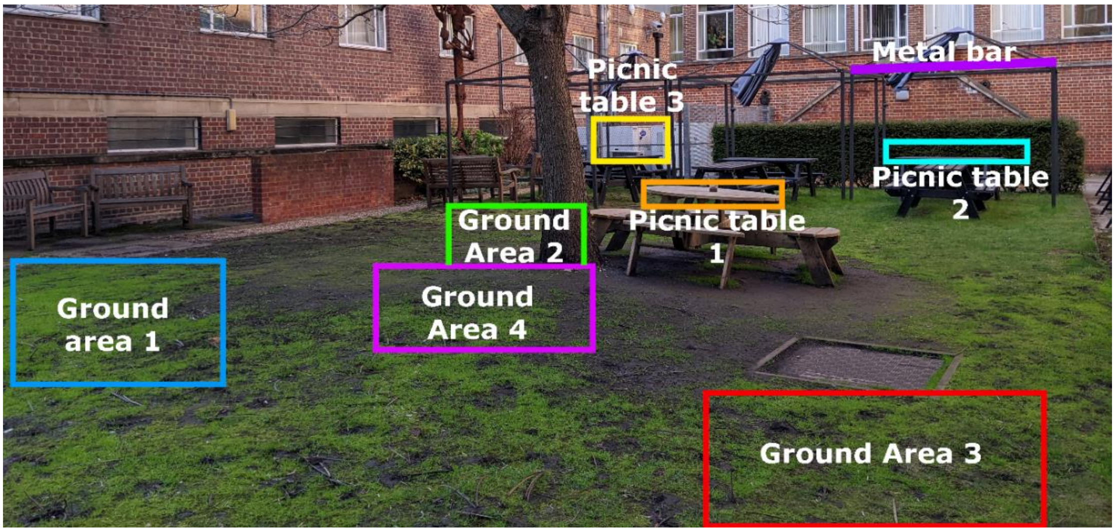

Green

Fig. 2 Objects and their locations in the outdoor area of the experiment. *The object locations for the outdoor environment. The top row shows the object locations for the Courtyard, and the bottom row for the Green. See Supplementary Table 1 for a list of which objects will be placed in which locations.*

Table 1 ***A table of the objects that will be used in the experiment, and which environment and location the objects will be placed in****. The trial order is the order if participants perform the blocks in the order 1, 2, 3, 4. However, the blocks will be counterbalanced so participants will always perform the trials in this order. For the congruency rating 1 is congruent and 2 is incongruent with the environment.*

Trial	Object	Environment	Location	Block	Congruency
1	Pen	Indoor	desk1, office 1	1	1
2	vacuum	Indoor	floor, Office1	1	2
3	holly	Indoor	desk1, office 2	1	2
4	grill	Indoor	seminar room floor 1	1	2
5	pillow	Indoor	chair, office 1	1	1
6	mug tankard	Indoor	table, seminar room	1	1
7	monitor	Indoor	desk1, office 1	1	1
8	binoculars	Indoor	table, seminar room	1	2
9	speaker	Indoor	table, office 3	1	1
10	coffee mug	Indoor	desk 2, office 1	1	1
11	hand saw	Indoor	desk 2, office 2	1	2
12	bird house	Indoor	wall1, corridor	1	2
13	shaker	Indoor	desk3, office 1	1	2
14	key	Indoor	table, seminar room	1	1
15	briefcase	Indoor	desk3, office 2	1	1
16	step stool	Indoor	floor, office 2	1	2
17	orchid	Indoor	desk1, office 2	1	1
18	spoon	Indoor	table, seminar room	1	1
19	beer	Indoor	desk1, office 1	1	2
20	slug	Indoor	floor, corridor	1	2
21	CD	Indoor	desk 2, office 2	2	1
22	computer	Indoor	table, office 3	2	1
23	moth	Indoor	wall2, corridor	2	2
24	funnel	Indoor	table, seminar room	2	2
25	office chair	Indoor	floor, office 2	2	1
26	scotch tape	Indoor	table, seminar room	2	1
27	waffle iron	Indoor	desk 2, office 2	2	2
28	French fries	Indoor	desk1, office 1	2	2
29	envelopes	Indoor	desk, seminar room	2	1
30	laptop	Indoor	table, office 3	2	1
31	headphones	Indoor	desk1, office 1	2	1
32	toaster	Indoor	table, office 3	2	2
33	plunger	Indoor	floor 2, seminar room	2	2
34	bowl	Indoor	table, seminar room	2	1
35	clamp	Indoor	table, office 3	2	2
36	food processor	Indoor	desk3, office 2	2	2
37	ruler	Indoor	desk 2, office 1	2	1
38	bracelet	Indoor	table, seminar room	2	1
39	hairbrush	Indoor	desk1, office 1	2	1
40	dryer	Indoor	floor 1, seminar room	2	2
41	bike helmet	Outdoor	picnic table 1, green	3	1
42	fork	Outdoor	picnic table 2, green	3	1
43	vase	Outdoor	picnic table 1, green	3	2
44	muffin	Outdoor	picnic table 3, green	3	1
45	tape	Outdoor	picnic table 1, green	3	2
46	Swiss army knife	Outdoor	picnic table 2, green	3	1
47	cat	Outdoor	ground area1, green	3	1

48	corkscrew	Outdoor	picnic table 2, green	3	2
49	crow	Outdoor	ground area1, green	3	1
50	grater	Outdoor	picnic table 3, green	3	2
51	squirrel	Outdoor	picnic table 1, green	3	1
52	bench	Outdoor	ground area1, green	3	1
53	sheep	Outdoor	ground area2, green	3	2
54	hanger	Outdoor	ground area 1, courtyard	3	2
55	sneakers	Outdoor	ground area 2, courtyard	3	2
56	cupboard	Outdoor	ground area 1, courtyard	3	2
57	bicycle	Outdoor	ground area 2, courtyard	3	1
58	sandal	Outdoor	ground area 1, courtyard	3	2
59	cigarette	Outdoor	ground area 2, courtyard	3	1
60	nut	Outdoor	ground area 1, courtyard	3	2
61	pine	Outdoor	picnic table 1, green	4	1
62	kingfisher	Outdoor	metal bar, green	4	2
63	cell phone	Outdoor	picnic table 2, green	4	2
64	lipstick	Outdoor	picnic table 3, green	4	2
65	dragonfly	Outdoor	picnic table 2, green	4	1
66	cabinet	Outdoor	ground area1, green	4	2
67	butterfly	Outdoor	ground area2, green	4	1
68	bush	Outdoor	ground area3, green	4	1
69	deer	Outdoor	ground area4, green	4	2
70	cap	Outdoor	picnic table 1, green	4	1
71	caterpillar	Outdoor	ground area1, green	4	1
72	thermos	Outdoor	picnic table 3, green	4	1
73	turkey	Outdoor	ground area1, green	4	2
74	pasta	Outdoor	picnic table 2, green	4	2
75	packing tape	Outdoor	picnic table 3, green	4	2
76	goose	Outdoor	ground area3, green	4	2
77	measuring tape	Outdoor	ground area 2, courtyard	4	2
78	loafer	Outdoor	ground area 1, courtyard	4	2
79	reindeer	Outdoor	ground area 3, courtyard	4	2
80	swan	Outdoor	ground area 1, courtyard	4	2

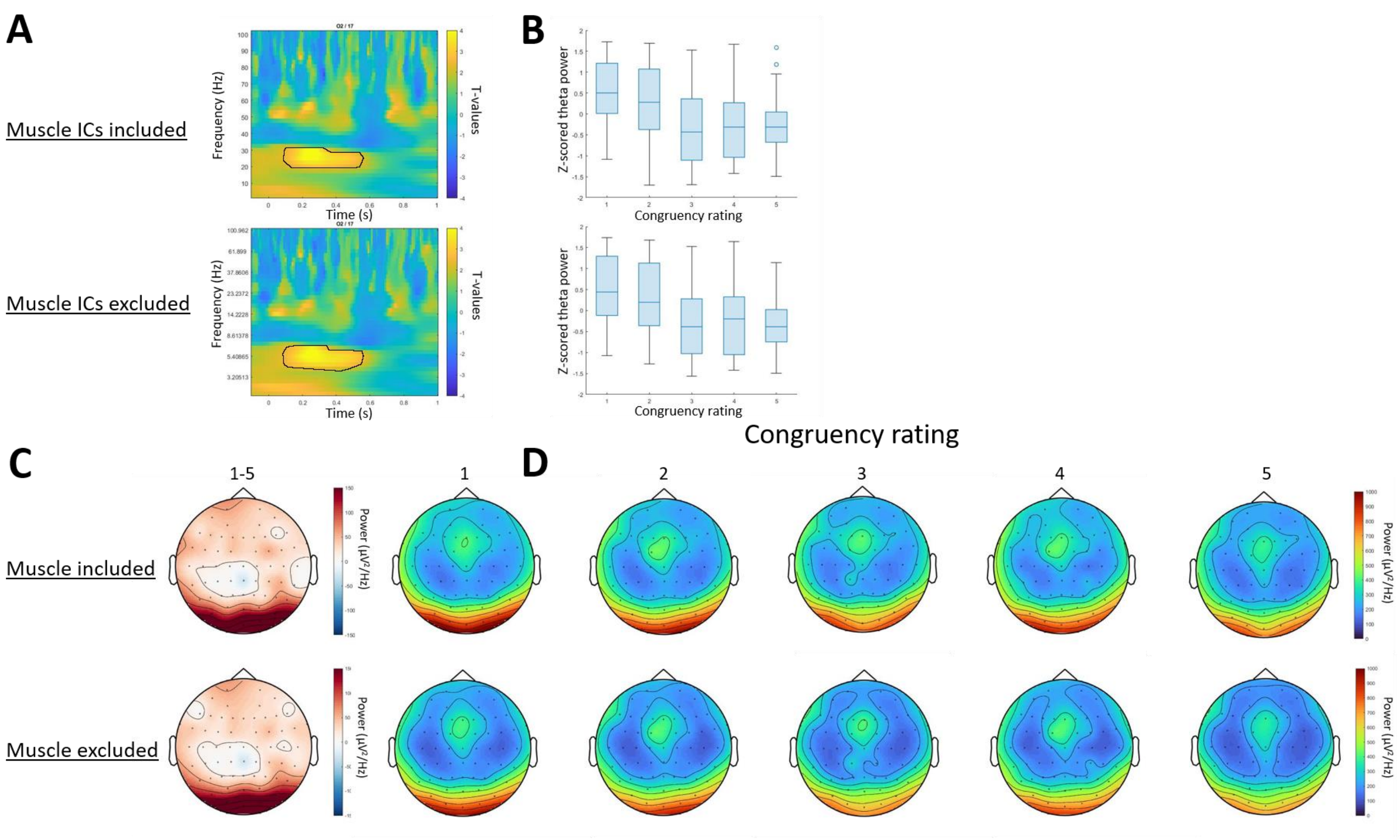

Fig. 3. Figures demonstrating the time-frequency analyses with muscle ICs included and excluded. (a) *T-values calculated across time and frequencies of the EEG data for electrode O2. The black line indicates the significant cluster for electrode O2. The top panel shows the results the results when muscle ICs are included and the bottom for when muscle ICs are excluded. (b) The z-scored theta power (4-7Hz) averaged across each of the five congruency ratings for each participant. The top panel shows the results the results when muscle ICs are included and the bottom for when muscle ICs are excluded. (c) The mean difference topography showing the difference in theta power between trials where objects were rated as highly incongruent (1) and highly congruent (5), between 200 and 500ms, averaged across participants. The top panel shows the results the results when muscle ICs are included and the bottom for when muscle ICs are excluded. (d) Topographies for the mean theta power (5-7Hz) between 200 and 500ms at each congruency rating sorted so that objects rated as highly incongruent (1) are on the left to objects rated as highly congruent (5) are on the right. The top panel shows the results the results when muscle ICs are included and the bottom for when muscle ICs are excluded.*
